# Entanglement, holonomic constraints, and the quantization of fundamental interactions

**DOI:** 10.1038/s41598-019-47844-8

**Published:** 2019-08-06

**Authors:** Salvatore Marco Giampaolo, Tommaso Macrì

**Affiliations:** 1 Division of Theoretical Physics, Rudjer Bošković Institute, Bijenčka cesta 54, 10000 Zagreb, Croatia; 2 Departamento de Física Teórica e Experimental and International Institute of Physics, Universidade Federal do Rio Grande do Norte, 59072-970 Natal-RN, Brazil

**Keywords:** Theoretical physics, Quantum mechanics

## Abstract

We provide a proof for the necessity of quantizing fundamental interactions demonstrating that a quantum version is needed for any non trivial conservative interaction whose strength depends on the relative distance between two objects. Our proof is based on a consistency argument that in the presence of a classical field two interacting objects in a separable state could not develop entanglement. This requirement can be cast in the form of a holonomic constraint that cannot be satisfied by generic interparticle potentials. Extending this picture of local holonomic constraints, we design a protocol that allows to measure the terms of a multipole expansion of the interaction of two composite bodies. The results presented in this work can pave the way for a study of fundamental interactions based on the analysis of entanglement properties.

## Introduction

More than a century after its birth, quantum mechanics is considered one of the physical theories that received the greatest experimental confirmation. It presents several aspects such as its intrinsically non-deterministic nature as well as quantum entanglement, that do not have a classical counterpart^1,2^. Thus, to provide a coherent picture of empirical evidence, great efforts were made to develop quantum versions of known classical theories, as for electromagnetism. Nowadays, electroweak and strong interactions, each one characterized by its own gauge group and force mediators, are elegantly described within the Standard Model of particle physics^3–6^.

In the present work we tackle the problem of the necessity of the quantization of fundamental interactions from a different point of view. We provide a general result that proves that any conservative interaction must have a quantized version, under the hypothesis that the effective interparticle potential depends on the relative distance between two objects but not on the derivative of any order of such a distance with respect to time. Starting from this assumption we show that the request that it does not create entanglement is equivalent to impose a holonomic constraint that cannot be satisfied for a generic setup. To enforce the consistency of the theory, we are then led to conclude that such interaction presents a quantum nature. Inspired by the discussion of the holonomic constraint, we propose a generalized spin-echo scheme^7^ to measure subleading terms in a multipole expansion of a generic conservative interparticle potential. This interferometric measurement is based on the efficient suppression of the phases of the dominant terms.

Our theorem provides an important and genuine result for all fundamental interactions except to gravity due to our current limited knowledge of gravitational interaction. We observe that, differently from the other forces, the quantization process of the gravity still presents several problems. As we explain below, a better understanding of gravitational interaction might be needed to make it fall under the hypotheses of our result. Even if different theories were developed^8^, including loop quantum gravity^9,10^ and string theory^11^, all of them are affected by some kind of problems^12–14^ and, up to now, a widely accepted quantum theory of gravity is still missing. A large debate rose about the fact that a quantum version of gravity is really needed or if, on the contrary, it must be considered an intrinsically classic interaction^14–19^ or an entropic force^20^. Over the last years, this discussion has been brightened by the publication of two independent works^21,22^. The authors of these papers suggest to use entanglement^2,23^, a quantity that is playing an ever-increasing role in very different fields ranging from metrology^24,25^ to quantum many-body systems^26–30^, to test the quantum nature of gravity experimentally. The key point of their proposal is that, if an interaction is intrinsically classical, and hence its evolution can be described by a Koopman-type dynamics^31^, it would not be able to generate entanglement between two massive objects. Indeed, from the point of view of quantum information theory, a classical field is equivalent to a classical channel that is unable to increase the entanglement between two systems at its end-points^32^. Therefore any evidence of entanglement between the two objects, generated by any conservative interactions, would be the smoking gun of its quantum nature. Such entanglement does not provide any information about the right quantum theory. However, it would prove that quantization is necessary to explain consistently the whole phenomenology associated with the experiments^33–35^.

**Figure 1:**
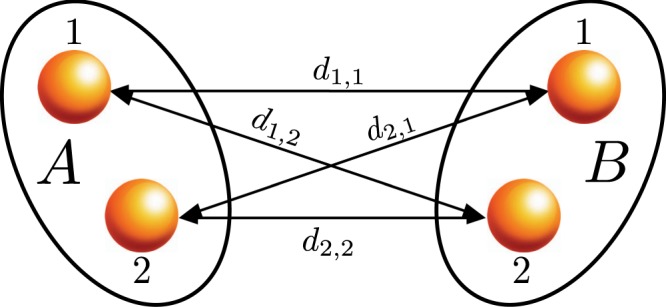
Sketch of the scheme used in the work. Two systems *A* and *B*, represented by the two ellipses, can be in two spatially separate states, named 1 and 2 represented by two gray circles. The interaction between the two systems is a function of the distance, and since it depends on the states occupied by the two systems, the interaction also becomes dependent on the state of the composite system.

The physical system which we consider in the present work is represented schematically in Fig. 1. Two physical objects, named *A* and *B* can be found in two different spatially separated internal states labeled 1 and 2. We assume that the interaction between the two bodies is mediated, over the distance, by a conservative force field whose classical or quantum nature is the subject of our analysis. Differently from all others physical characteristics, such as mass, charge, flavor etc. that we assume time-independent, the relative distances between the different states of *A* and *B* can be functions of time. The reason to consider spatially separated states is to create a state-dependent interaction. More general implementations of potentials depending on the internal states will be considered below.

## Results

For the setup of Fig. 1 the Hamiltonian of the system reads

1 $$\begin{array}{rcl}\hat{H} & = & f({d}_{1,1}(t))|1,1\rangle \langle 1,1|+f({d}_{1,2}(t))|1,2\rangle \langle 1,2|\\  &  & +\,f({d}_{2,1}(t))|2,1\rangle \langle 2,1|+f({d}_{2,2}(t))|2,2\rangle \langle 2,2|.\end{array}$$

Here *f* stands for the interaction energy that is a real function of the distance ${d}_{\alpha ,\beta }(t)$ that depends on the state *α*, *β* of the first *A* (*α*) and the second *B* (*β*) object as well as on time *t*. Defining, for every single object, the operators ${\sigma }_{i}^{z}=|{1}_{i}\rangle \langle {1}_{i}|-|{2}_{i}\rangle \langle {2}_{i}|$ we have that the Hamiltonian in eq. (1) can be rewritten as

2 $$\begin{array}{rcl}\hat{H} & = & {g}_{1}{{\bf{1}}}_{A}\otimes {{\bf{1}}}_{B}+{g}_{2}{\sigma }_{A}^{z}\otimes {{\bf{1}}}_{B}+{g}_{3}{{\bf{1}}}_{A}\otimes {\sigma }_{B}^{z}+{g}_{4}{\sigma }_{A}^{z}\otimes {\sigma }_{B}^{z};\\ \hat{H} & = & \sum _{i}\,{g}_{i}{\hat{H}}_{i},\end{array}$$

where **1**_*α*_ is the identity operator on the physical object *α* and the coefficient *g*_*j*_ are linear combinations of *f*(*d*_*α*,*β*_).

Let us now consider that, at *t* = 0, our system is in a state that is a tensor product of two states, each one of them defined on a single object. Each one of these two states is a linear superposition of the state of the objects, i.e. ${c}_{\alpha }^{(1)}|{1}_{\alpha }\rangle +{c}_{\alpha }^{(2)}|{2}_{\alpha }\rangle$, $\alpha =A,B$. For sake of simplicity we assume, in both cases, that the linear superposition is symmetric and real, i.e. $|{\psi }_{i}(0)\rangle =\frac{1}{\sqrt{2}}(|{1}_{i}\rangle +|{2}_{i}\rangle )$ and $|{\rm{\Psi }}(0)\rangle =|{\psi }_{A}(0)\rangle \otimes |{\psi }_{B}(0)\rangle$.

The initial state $|{\rm{\Psi }}(0)\rangle$ evolves dynamically as $|{\rm{\Psi }}(t)\rangle =\hat{U}(t)|{\rm{\Psi }}(0)\rangle$, where $\hat{U}(t)=\exp (\,-\,\iota \hat{H}t)$ is the time evolution operator and $\hat{H}$ is defined in eq. (2). Since the Hamiltonian is the sum of a commuting set of operators, $\hat{U}(t)$ can be factorized into the product of four unitary operators each one depending on one single term of the Hamiltonian, i.e. $\hat{U}(t)={\prod }_{i}\,{\hat{U}}_{i}(t)$ where ${\hat{U}}_{i}(t)=\exp (\,-\,\iota {g}_{i}{\hat{H}}_{i}t)$.

It is easy to see that the rise of the entanglement in the system depends only on the action of ${\hat{U}}_{4}(t)$. In fact, ${\hat{U}}_{1}(t)$ is proportional to the identity operator and hence it contributes to the evolution only with a global phase factor. On the other hand ${\hat{U}}_{2}(t)$ and ${\hat{U}}_{3}(t)$, being ${\hat{H}}_{2}={\sigma }_{A}^{z}\otimes {{\bf{1}}}_{B}$ and ${\hat{H}}_{3}={{\bf{1}}}_{A}\otimes {\sigma }_{B}^{z}$, act as local operators and hence are unable to create entanglement. Therefore if entanglement between *A* and *B* is present it has to be related to the action of ${\hat{U}}_{4}(t)=\exp (\,-\,\iota {g}_{4}t\,{\sigma }_{A}^{z}\otimes {\sigma }_{B}^{z})$. Hence, for what concerns the entanglement properties, the state $|{\rm{\Psi }}(t)\rangle =\hat{U}(t)|{\rm{\Psi }}(0)\rangle$ is equivalent to the state ${\hat{U}}_{4}(t)|{\rm{\Psi }}(0)\rangle$ that is

3 $${\hat{U}}_{4}(t)|{\rm{\Psi }}(0)\rangle =\frac{{e}^{-\iota \varphi }}{2}({e}^{2\iota \varphi }|1,1\rangle +|1,2\rangle +|2,1\rangle +{e}^{2\iota \varphi }|2,2\rangle ),$$

where the phase $\varphi$ is given by

4 $$\begin{array}{rcl}\varphi  & = & {\int }_{0}^{t}\,{g}_{4}(\tau )d\tau \\  & = & {\int }_{0}^{t}\,[f({d}_{1,1}(\tau ))+f({d}_{2,2}(\tau ))-f({d}_{1,2}(\tau ))-f({d}_{2,1}(\tau ))]d\tau .\end{array}$$

For a state described by eq. (3) the entanglement can be quantified by the concurrence ${\mathscr{C}}$^36,37^. In this specific case it equals ${\mathscr{C}}=|\,\sin (2\varphi )|$. However, in agreement with well-known quantum information results^2,32^, and the conclusions of ref.^22^ for a Koopman-type dynamics^31^, an intrinsically classical interaction cannot generate entanglement. Hence, in the case of a classic Koopman-type interaction, we must have that ${\mathscr{C}}=0$ for all possible set of the parameters of the system. Since both the time-dependent distances and the integration time are arbitrary, to ensure that ${\hat{U}}_{4}(t)|{\rm{\Psi }}(0)\rangle$ remains separable for any time $t > 0$, we have to require that the function inside the integral in eq. (4) must vanish for all possible sets of distances, i.e.

5 $$f({d}_{1,1})+f({d}_{2,2})-f({d}_{1,2})-f({d}_{2,1})=0.$$

All the interactions for which at least one physically achievable setup exists that does not satisfy eq. (5) can generate entanglement. Then, a formulation of the fundamental theory based on a classical mediating field leading to the *effective* nonlocal interaction $f({d}_{\alpha ,\beta })$ would prove to be inconsistent with entanglement generation. It is important to underline that eq. (5) was derived within the hypothesis that the interaction depends only on the distance and not on a generic derivative of the relative position of the states of the two objects.

In the Methods section we will provide the proof that any non-trivial conservative interaction, i.e. any interaction which amplitude is non independent by the distance, violates the condition in eq. (5). Consequently all of them need a quantum theory to explain coherently all the different physical phenomena.

As an example we consider an interparticle potential $f(d)=\lambda \,{d}^{-\alpha }$ where *λ* is the coupling constant and *α* the power-law exponent of the decay of the interaction with distance. For sake of simplicity we choose: (1) the two quantum objects *A* and *B* at rest in the same inertial reference system; (2) the four states on the same line; (3) the distance between the two states of the same object equal to be *δ*_*x*_. Accordingly with these choice we have that the four distances are ${d}_{1,1}=x$, ${d}_{2,1}={d}_{1,2}=x+{\delta }_{x}$, ${d}_{2,2}=x+2{\delta }_{x}$ and ${\delta }_{x}\ll x$. With this assumption we have that the phase $\varphi (t)$ varies linearly with *t* and it is given by

6 $$\varphi (t)=\lambda \,t\frac{\alpha (\alpha +1)\,{\delta }_{x}^{2}}{{x}^{2+\alpha }}.$$

The phase in eq. (6) vanishes only in the trivial case in which ${\delta }_{x}=0$, i.e. if the distance between the two states of the same physical objects goes to zero. Indeed, in such case, the interactions becomes independent on the states and the evaluation only rises a global phase factor without inducing a creation of non-vanishing entanglement.

We now discuss the second point of our work. In general, when studying the interaction between two bodies, it is often useful to describe their effective interaction potential in terms of a multipole expansion. Each multipole of the series contains an angular dependence as well as an inverse power of the distance among a (composite) body and a reference point or another body. This standard procedure is widely used in problems involving gravitational systems of masses or electric and magnetic distributions of charges and currents. The calculation and measurement of multipole moments to characterize the interaction potential is relevant to the experimental setups mentioned above. To this purpose, we propose to make use of the analysis of the entanglement to estimate the strength of the subdominant terms of the potential.

**Figure 2:**
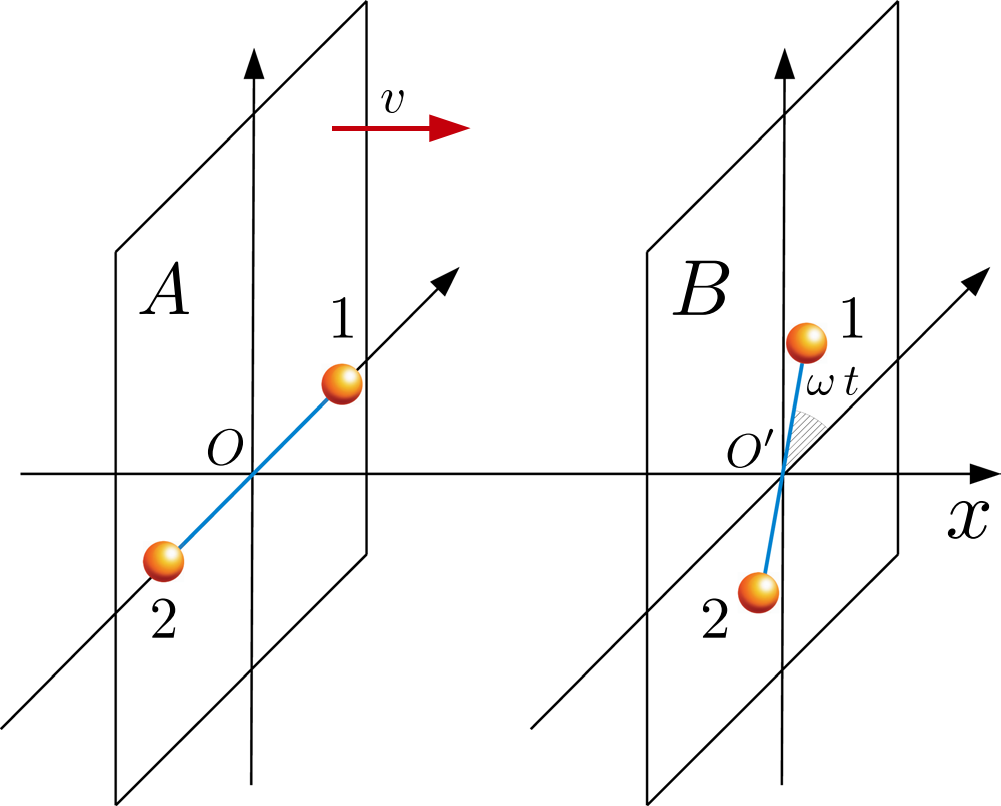
Sketch of the proposed experimental protocol. The two objects lie on two parallel planes whose relative distance varies in time with a constant velocity *v*. The object *A* is at rest while *B* rotates with an uniform angular velocity $\omega$ around the center of the segment joining the positions associated with its two internal states.

As an example we consider a Laurent expansion for the function *f*(*d*), neglecting for simplicity angular dependence of the multipoles,

7 $$f(d)=\sum _{n}\,{f}_{n}(d)=\sum _{n}\,{c}_{n}{d}^{-n}$$

where each term has a definite power-law dependence on distance. Also in this case a straightforward generalization to interaction potentials depending on the relative coordinates and not only on the relative distance can be easily done. To increase the signal-to-noise ratio we design a protocol in a way that the entanglement generated by the main terms vanishes. Focusing on the *n*-th term of the potential, assuming $|{f}^{(n)}(d)|\gg |{f}^{(n+1)}(d)|$, and defining

8 $${\varphi }^{(i)}={c}_{n}\,{\int }_{0}^{t}\,[\frac{1}{{d}_{1,1}{(t)}^{n}}+\frac{1}{{d}_{2,2}{(t)}^{n}}-\frac{1}{{d}_{1,2}{(t)}^{n}}-\frac{1}{{d}_{2,1}{(t)}^{n}}]d\tau ,$$

we have to set ${\sum }_{i=1}^{n-1}\,{\varphi }^{(i)}=0$. We apply this scheme to the case of the first correction to a potential whose leading term is ${f}^{(1)}(d)={c}_{1}\,{d}^{-1}$. The first subleading term then reads ${f}^{(2)}(d)={c}_{2}\,{d}^{-2}$. We then consider a system as in Fig. 2: (1) Two identical objects lie on two parallel planes; (2) The distance between the two internal states of each object is *x*_0_; (3) The line joining the midpoints of the center of mass of each body is perpendicular to the planes; (4) The inter-plane distance reduces at constant rate *v*, i.e. it follows the law *L* − *vt*; (5) The object *B* rotates on its plane with uniform angular velocity $\omega$ around the midpoint of the segment passing through the two states. (6) We prepare the initial state with the linear and angular velocity at $t=0$ and then we let them evolve only under the effects of their relative interaction Hamiltonian. In agreement with these assumptions, the state dependent distances become

9 $$\begin{array}{lllll}{d}_{11}(t) & = & {d}_{22}(t) & = & {[{(L-vt)}^{2}+{x}_{0}^{2}{\sin }^{2}(\omega t/2)]}^{1/2},\\ {d}_{12}(t) & = & {d}_{21}(t) & = & {[{(L-vt)}^{2}+{x}_{0}^{2}{\cos }^{2}(\omega t/2)]}^{1/2}.\end{array}$$

Said t* the optimal time at which ${\varphi }^{(1)}=0$ we have that, if *v*, $\omega \ne 0$ then ${\varphi }^{(2)}(t)\ne 0$. As a consequence of point 6) the system, during its evaluation can be considered a closed one. Hence the evolution is unitary and there is no entropy flow from the system to the rest of the universe. Therefore we are in the same situation that we have faced in the first part of this section and we can apply to the situation represented in Fig. 2 all the previous results.

**Figure 3:**
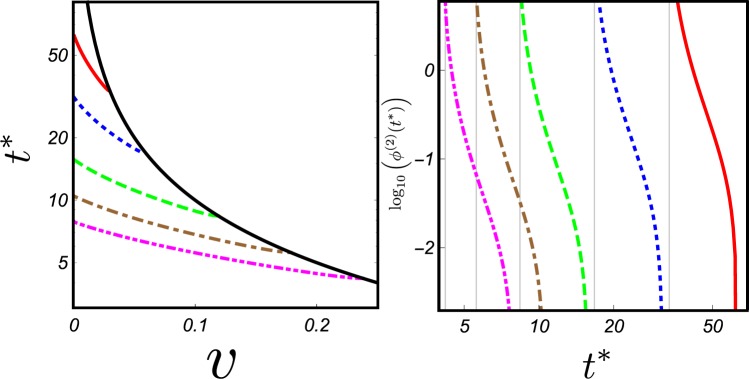
Optimal time t* and dynamical phase ${\varphi }^{(2)}({t}^{\ast })$. (**a**) t* as function of the velocity *v* for different values of $\omega$. (**b**) ${\mathrm{log}}_{10}\,{\varphi }^{(2)}({t}^{\ast })$ for the t* evaluated in the left plot for several value of $\omega$. For both plots we set: $\omega =0.05$ (red solid); $\omega =0.1$ (blue dotted line); $\omega =0.2$ (green dashed line); $\omega =0.3$ (brown dot-dashed line); $\omega =0.4$ (magenta dot-dot-dashed line). The black line in the left figure displays the time $\bar{t}=L/v$ in which the two physical objects collide. The gray vertical grid lines in the right plots indicate, for each $\omega$ the time at which ${t}^{\ast }=\bar{t}$. In the numerical simulation we assumed *δx*_0_ = 0.1 *L* and we normalized all quantities in such a way that *c*_2_ = 1.

In the left panel of Fig. 3 we plot t* as function of *v* for different $\omega$ whereas in the right one we show the behavior of the phase ${\varphi }^{(2)}({t}^{\ast })$ induced by the sub-leading term ∝ *d*^−2^. As general result we can see that ${\varphi }^{(2)}({t}^{\ast })$ increases decreasing *v* and/or $\omega$ and tends to diverge when t* goes towards $\bar{t}=L/v$, i.e. when the time in which ${\varphi }^{(1)}$ vanishes coincides with the time at which the two physical objects collide. Bringing t* versus $\bar{t}$ and reducing *v* and $\omega$ we maximize the total integration time and we enter in a region of the space in which the subleading term is more relevant. Notably this protocol allows to measure the dynamical phase in a much faster time interval than the one would get upon keeping interparticle distances fixed. This is of utmost relevance for any possible experimental realization. Indeed decoherence effects or losses reduce, over time, the coherent superposition in a statistical mixture in which all entanglements vanish in any realistic device^38–40^.

## Discussion

In this work we proved that every non-trivial conservative potential will generate entanglement between two physical objects. Hence, for all conservative interactions a quantized description must hold. The proof is based on a holonomic constraint that must be satisfied at any time *t* if the mediating field were classical for it not to generate entanglement. This requirement, however, would rule out all non-trivial interactions. The scheme discussed in the first part can be easily generalized to include more complex dependence of the potential on the relative distance or state-dependent interactions.

At this point it is legitimate to ask what our result entails for the case of the gravitational interaction. To date no one knows what the exact form of the gravitational interaction is at microscopic distances, or whether the interaction ceases to be conservative or if takes the form discussed in this work. Therefore, at least at the fundamental level we cannot conclude whether gravitational interactions falls within the range of validity of our theorem. If gravity is, in some region of parameter space, describable as a conservative force that falls within the hypotheses of our theorem, then in that regime it must admit a quantum theory. It seems then plausible that quantization is needed anywhere. To shed some light an experimental test has been proposed using levitated diamond nanocrystals with nitrogen-vacancy (NV) centers in high vacuum as discussed in^21,41^. These proposals focussed on the coherent superposition of spatially separated states of massive objects. However, as mentioned above, the basic requirement is to generate a state dependent interaction. This can be achieved even in the absence of spatial separation of the internal states. For example one can consider particles in which the internal states are characterized by different masses, as neutrinos^42^. Alternatively one can take into account Rydberg atoms in microtraps which can be optically manipulated with high precision^43^. For this system the two internal states would correspond to either the ground state and one highly excited Rydberg level or two excited states interacting electromagnetically^44–46^.

In the second part we have shown how it is possible to measure the various terms of a multipole-like Laurent expansion of a conservative interaction potential via the measurement of the entanglement between the two objects. This scheme is based on a proper manipulation of spatial and internal degrees of freedom of a two-body system during the interaction time. We notice that this protocol applies well to both electromagnetic interactions, i.e. to mass or charge/current distributions and the gravitational interactions if the effective low-energy nonrelativistic theory can be described by a position and state-dependent potentials in the way assumed in our derivation. Moreover for gravity it is possible, at least in principle, to use such kind of test to investigate the presence of a Yukawa-type corrections at the Newtonian potential associated to the presence of a graviton with a non vanishing mass^47–49^ or of extra dimensions with a finite radius of compactification^50^. However, the application of this scheme for such terms would not produce observable effects. Indeed the relativistic corrections are proportional to ${\ell }_{cl}/r$, where ${\ell }_{cl}=Gm/{c}^{2}=7.4\cdot {10}^{-42}\,{\rm{m}}$ for a mass $m={10}^{-14}\,{\rm{kg}}$. Quantum corrections are proportional to ${({\ell }_{P}/r)}^{2}$, where ${\ell }_{P}=1.6\cdot {10}^{-35}\,{\rm{m}}$ is the Planck length.

## Methods

### Holonomic constraint and conservative interactions

We prove that eq. (5)

10 $$f({d}_{1,1})+f({d}_{2,2})-f({d}_{1,2})-f({d}_{2,1})=0.$$

is violated by all non-trivial conservative interactions. Let us define the vector **x** in ${{\mathbb{R}}}^{\otimes 12}$ that denotes the four coordinates in real space of the internal states in Fig. 1 and the function $h({\bf{x}})=f({d}_{1,1})+f({d}_{2,2})-f({d}_{1,2})-f({d}_{2,1})$. Equation (5) can then be rewritten as $h({\bf{x}})=0$. Such condition, as the interaction is conservative, expresses a holonomic constraint of the corresponding dynamics, as it depends just on the vector of the coordinates but not on the velocities or any higher order derivative with respect to time. We name ${\mathcal L}$ the set of points ${\bf{x}}\in {{\mathbb{R}}}^{\otimes 12}$ that satisfy the holonomic constraint. It is immediate to note that the trivial interaction $f(d)=const$ fulfills the constraint for all possible configurations and hence ${\mathcal L} \equiv {{\mathbb{R}}}^{\otimes 12}$. Besides, for a generic interaction one has ${\mathcal L}$ is not an empty set. For example, the subset of configurations which fulfill ${d}_{1,1}={d}_{1,2}$ and ${d}_{2,1}={d}_{2,2}$ always satisfies $h({\bf{x}})=0$. However, for a generic position dependent interaction, we have that ${\mathcal L}$ ≡ ${{\mathbb{R}}}^{\otimes 12}$. Indeed, let us consider ${{\bf{x}}}_{0}\in  {\mathcal L}$ and $\hat{{\bf{n}}}$*δ* a vector of modulus *δ* and direction $\hat{{\bf{n}}}$ defined in ${R}^{\otimes 12}$. If the function *f*(*d*) is analytic also *h*(**x**) will be analytic and assuming *δ* small enough we can write ${h({{\bf{x}}}_{0}+\hat{{\bf{n}}}\delta )=\tfrac{\partial h}{\partial \hat{{\bf{n}}}}|}_{{\bf{x}}={{\bf{x}}}_{0}}\delta$. But if ${\mathcal L} \equiv {{\mathbb{R}}}^{\otimes 12}$, also ${{\bf{x}}}_{0}+\hat{{\bf{n}}}\delta$ should lay in ${\mathcal L}$ and, since *δ* ≠ 0 we must have ${\tfrac{\partial h}{\partial \hat{{\bf{n}}}}|}_{{\bf{x}}={{\bf{x}}}_{0}}=0$ for all $\hat{{\bf{n}}}$ and all **x**_0_. This result can be generalized to any order of approximation of the interaction potential and, therefore, to have that ${\mathcal L} \equiv {{\mathbb{R}}}^{\otimes 12}$ the interaction potential *f*(*d*) must be constant all over the space. We then conclude that a non-trivial interaction induces entanglement whenever **x** ∉ ${\mathcal L}$.

## Data Availability

Data are available upon request. Requests should be addressed to either author.
